# Sliding properties of anodized aluminium alloy tested in water and hydraulic oil

**DOI:** 10.1038/s41598-026-39681-3

**Published:** 2026-02-14

**Authors:** Ana Trajkovski, Jan Bartolj, Nejc Novak, Jan Pustavrh, Mitjan Kalin, Franc Majdič

**Affiliations:** 1 Faculty of Mechanical Engineering, Laboratory for Fluid Power and Controls, University of Ljubljana, Aškerčeva cesta 6, 1000 Ljubljana, Slovenia; 2 Faculty of Mechanical Engineering, Laboratory for Tribology and Interface Nanotechnology, University of Ljubljana, Aškerčeva cesta 6, 1000 Ljubljana, Slovenia

**Keywords:** Water hydraulics, Aluminium alloy, Anodization, Wear, Friction, Oil, Engineering, Materials science

## Abstract

Long operation and efficiency of hydraulic valves are often limited by the construction and design of the chosen valve. Faster switching of control valves can also be achieved by reducing the mass of the moving spool through the use of different materials. In addition to material and design optimization, current research and development in advanced hydraulic systems increasingly emphasise the use of environmentally acceptable lubricants. This study investigates alternative material pairs for a water hydraulic spool valve. Specifically, natural aluminium alloy (EN AW-6082) and anodized aluminium alloy were tested in water. The results were compared with reference measurements in hydraulic oil ISO VG46 and measurements of the conventional valve spool material, nitrided C45 steel. The tribological tests were performed in ball-on-disk reciprocating motion for 90 min, using load of 50 N and a stroke length 2.4 mm. The tests were performed at two sliding frequencies of 10 Hz and 40 Hz, resulting in linear speed of 0.05 m/s and 0.2 m/s. The results of the study show that anodized aluminium alloy has lower coefficient of friction (0.36) and reduced specific wear (1.49 × 10^− 6^ mm^3^/Nm) compared to natural aluminium alloy in water at lower sliding speed, with both measured values becoming comparable to the values measured for nitrided C45 steel. However, at higher sliding speeds the anodized aluminium alloy has even lower coefficient of friction (0.34) and gives better results in measured specific wear compared to natural aluminium alloy, but not at the same order of magnitude as measured for nitrided C45 steel. Under oil lubricated conditions, the tested sliding speed had no influence on the improved tribological properties of anodized aluminium alloy which were comparable to the measured values for nitrided C45 steel.

## Introduction

One of the greatest challenges of sustainable hydraulics is increasing the efficiency of hydraulic components. One of the possible approaches is trying to lower the coefficient of friction and specific wear in the contacts of hydraulic components involved. The results of resent research imply that the use of innovative materials and design solutions could reduce energy losses due to friction and processing up to 40 % in 15 years^1^. Of all hydraulic components, the control components are the most critical, as they have a major impact on the overall energy efficiency of hydraulic systems^2^. Valves determine both the direction and the volumetric flow rate of the working fluid. Compared to expensive servo control technology, digital technology uses conventional valves to actively control the system^3–5^. Digital technology is a potential solution for replacing traditional servo control technology in the era of “Industry 4.0”^6^. Digital valves mainly consist of conventional on/off valves, primarily a seat-type or alternatively spool-type valves^7^. Digital 2/2-way valves with sliding control spools are of particular interest, as their high switching frequencies enable precise and dynamic system regulation.

Faster switching, higher flow rates and greater energy efficiency can also be achieved by reducing the mass of the valve’s moving element using various innovative materials and technologies^3,7^. Aluminium alloys are often used to reduce the product mass, although now days different polymer composites are also becoming very popular^8,9^. Due to aluminium low specific gravity, low weight, high thermal and electrical conductivity and easy availability and machinability^10,11^, aluminium alloys are the most commonly used metal. These materials are commonly used in in the automotive, aviation, marine, military, electronic, food and pharmaceutical industries^12–16^. However, due to aluminium low hardness and sensitivity to abrasive wear, the use of these materials is often limited to applications with low friction and lower loads^17,18^. In order to increase the surface hardness of aluminium parts, various surface protection processes have been developed, such as conventional and hard anodising^18^, micro-oxidation (plasma electrolytic oxidation)^17^ and thermal hardening with oxygen (high velocity oxygen spraying - HVOF)^19^. In conventional anodising, aluminium oxides up to a thickness of 25 μm are usually produced. With hard anodising, similar to HVOF^19^, oxides thicknesses between 25 and 150 μm ^18^ are achieved. Such procedures enable increased surface hardness by a factor of 5 to 8 ^11^ and lower friction coefficient and wear rate about 3–100 times^16^. The process itself and the anodizing parameters (acid type, electrolyte concentration, current density, anodization time^16,20^ significantly influence the wear - corrosion and abrasion resistance of the aluminium alloy, and thus have been investigated intensively^21,22^. Commonly mechanical properties of anodized layers are evaluated in literature^11,23,24^. Despite the wide use of aluminium and its alloys in various fields of industry, the tribological properties are mostly investigated in the dry state^11,16,18,20,22,25–27^, and in some cases in hydraulic oil^28,29^ and recently vegetable oil^30^. However anodized aluminum alloys are traditionally used in hydraulic systems to improve wear resistance, corrosion resistance, and lubricity, particularly in components like cylinders, spool valves, and manifolds where oil lubricated conditions are expected.

A major weakness of traditional hydraulics is the risk of environmental pollution in the event of oil leakage, which cannot be controlled, and the waste oil cannot be effectively reused^31^. Current United Nations directives aligned with European Union and national emission regulations, have introduced limitations on the use of conventional oil-based systems. As a result, environmentally acceptable lubricants (EALs) are becoming mandatory in sectors such as maritime transport and hydropower. In applications where the risks associated with critical oil leakage (contamination, toxicity, or fire) including nuclear engineering, coal mining, steel production, firefighting, food and beverage processing EAL are also becoming important. Similar trends are observed in agriculture, forestry, mobile machinery, food-processing operations, and the pharmaceutical industry^31^.This growing regulatory pressure, combined with increased environmental awareness, has stimulated renewed interest in alternative solutions. Vegetable oils, ionic liquids^32–34^ and water with different additives (glycerol^35,36^, nanoparticles^37–39^ etc.) are often investigated as potential alternatives in hydraulics. One of the oldest and simplest hydraulic fluids (from first hydraulic pump around 270 BC and first hydraulic press in 1795) is water. Water remained the key hydraulic drive medium until the early 20th century, when it was gradually replaced by hydraulic oils due to technical limitations. Water hydraulics is an interesting alternative to oil hydraulics as it uses a clean, environmentally friendly and non-flammable medium.

However, water has distinct disadvantages – primarily low viscosity (even 100 times lower than oil), limited lubricating abilities, and chemical reactivity that can cause corrosion, and the risk of cavitation. Modern materials and technologies offer opportunities for effective management of these disadvantages. Thus, the availability of good control valves, which are essential components of hydraulic systems, is limited for water hydraulics^40^. The problem of precisely establishing small clearances between the moving elements of the valve. This is particularly difficult when the working medium is water. This severely limits the use of water hydraulics and increases the product price. The cost of stainless-steel components (commonly used material in water-hydraulics) is currently 30 to 400 % higher than the cost of standard oil hydraulic elements (commonly made of steel alloys, cast iron, aluminium alloys, bronze)^41,42^. While advanced materials like high-performance plastics and ceramics can reduce wear, their brittleness, manufacturing complexity, and sensitivity to water cleanliness limit practical application. There is not a lot of water hydraulic valves on the market^43^. There are even less water-hydraulic spool-type valves, commonly for lower working pressures (up to 25 MPa) and lower flow rates (up to 80 l/min)^43^. Combined with maintenance and cost constraints, these issues hinder the broader adoption of spool valves in water hydraulics.

In our study, a standard material pair of the original spool type on/off valve was compared to two alternative tribological pairs for water hydraulics. The sliding contact between stainless steel valve body and nitrided C45 valve spool was analysed, as reference sliding contact. As an alternative material for sliding spool an aluminium alloy (EN AW 6082) and anodized aluminium alloy were tested. The tribological properties of the selected material pairs were compared under water and hydraulic oil ISO VG46 lubricated conditions in reciprocating ball-on-disk experiments. Since operating frequency is important factor, the experiments were performed at two different sliding speeds.

## Materials and methods

### Sample preparation

Disc specimens of C45 steel and EN AW-6082 aluminium alloy, were made from a 30 mm diameter bars and cut to a thickness of 6 mm. All discs were subsequently grounded in multiple stages using a RotoForce-3 polishing and sample preparation device to a final roughness of Ra = 0.1 μm. We aimed for a surface roughness (Ra) of 0.1 μm to ensure that the tribological test conditions closely matched those of a real valve, who’s housing typically exhibits the same roughness level. The C45 steel discs were subsequently treated with a nitriding process to harden the surface (underwent the plasma nitrocarburizing, at temperature 500–520 °C and pressure 3 mbar; for the period of 12 h, with layer thickness of 30 μm). Half of natural aluminium alloy discs were anodized (sulfuric acid concertation 30–40 Vol %, used voltage 17–20 V; at temperature 16–17 °C; for the period of 45 min; with layer thickness 10–20 μm).

The hardness of the disc specimens was measured in accordance with DIN 50,133 standard, using a Vickers microhardness tester Leitz - Miniload 2. Hardness impressions were generated using a pyramidal indenter under a test load of 2 N, for 10 s. The material properties of all used discs are given in Table 1 below.

For the reciprocating ball-on-disc tribological tests, a commercially available hardened bearing ball made of AISI 440 C stainless steel, with a diameter of 25 mm, was used as the counter body.

**Table 1:** Mechanical properties of used materials.

Material	Denisty ρ [g/cm^3^]	Modulus of elasticity [GPa]	Tensile strength [MPa]	Hardness [HV]
AISI 440-C	7.7	210	1900–2000	~ 695
Nitrided C45	7.85	205	700–850	~ 630
Al EN AW-6082	2.7	70	205	~ 131,5
Anodized Al EN AW-6082	2.7	70	205	~ 200–250

The properties of the lubricants used in this study were determined using an Anton-Paar-SVM 3001 automatic viscometer. Demineralized water (density 0.997 g/cm^3^, kinematic viscosity 0.89 mm^2^/s at 25 °C) was selected as primary test lubricant. For comparative purposes, a conventional mineral hydraulic oil, ISO VG46 (density 0.861 g/cm^3^, kinematic viscosity 100 mm^2^/s at 25 °C), was also evaluated.

### Tribological measurements

The tribological measurements were performed using a Cameron Plint TE77 high-frequency ball-on disc tribometer. Prior to each experiment, all components in contact: the specimens, grippers, steel ball and the bath were thoroughly cleaned in ethanol and subsequently dried in an air stream. The specimen was then placed in the bath, secured with the grippers, and fully immersed in the selected lubricant before testing. All experiments were conducted at a room temperature of 25 *±* 1 °C, which was monitored with the temperature probe positioned at the bottom of the lubricant bath.

A normal load of 50 N was applied to the stainless-steel bearing ball, a value determined from preliminary measurements to ensure steady-state friction and wear for all tested material pairs. The corresponding maximum Hertzian contact pressures were 930 MPa for the steel ball–steel disc pair and 595 MPa for the steel ball–aluminium alloy (EN AW-6082) pair.

The stroke length was 2.4 mm, corresponding to the valve spool displacement occurring during position switching in CETOP 3 proportional valves. This value also represented the minimum stroke length available with the tribometer employed in the experiments. The tests were performed for 90 min, based on preliminary tests, to achieve steady-state friction and wear for all the samples. Two reciprocating frequencies were used: 10 Hz, corresponding to a sliding speed of 0.05 m/s, and 40 Hz, corresponding to a sliding speed of 0.19 m/s. These conditions resulted in 54,000 and 216,000 cycles (a total sliding distances of 259.2 m and 1036.2 m, respectively).

The coefficient of friction was recorded continuously throughout the experiment, for each individual cycle. For each lubricant (demineralized water and ISO VG46 hydraulic oil) and each material pair, three independent measurements were conducted. The mean value of the stabilized coefficient of friction was used in the subsequent analysis.

### Wear analyses

**Fig. 1:**
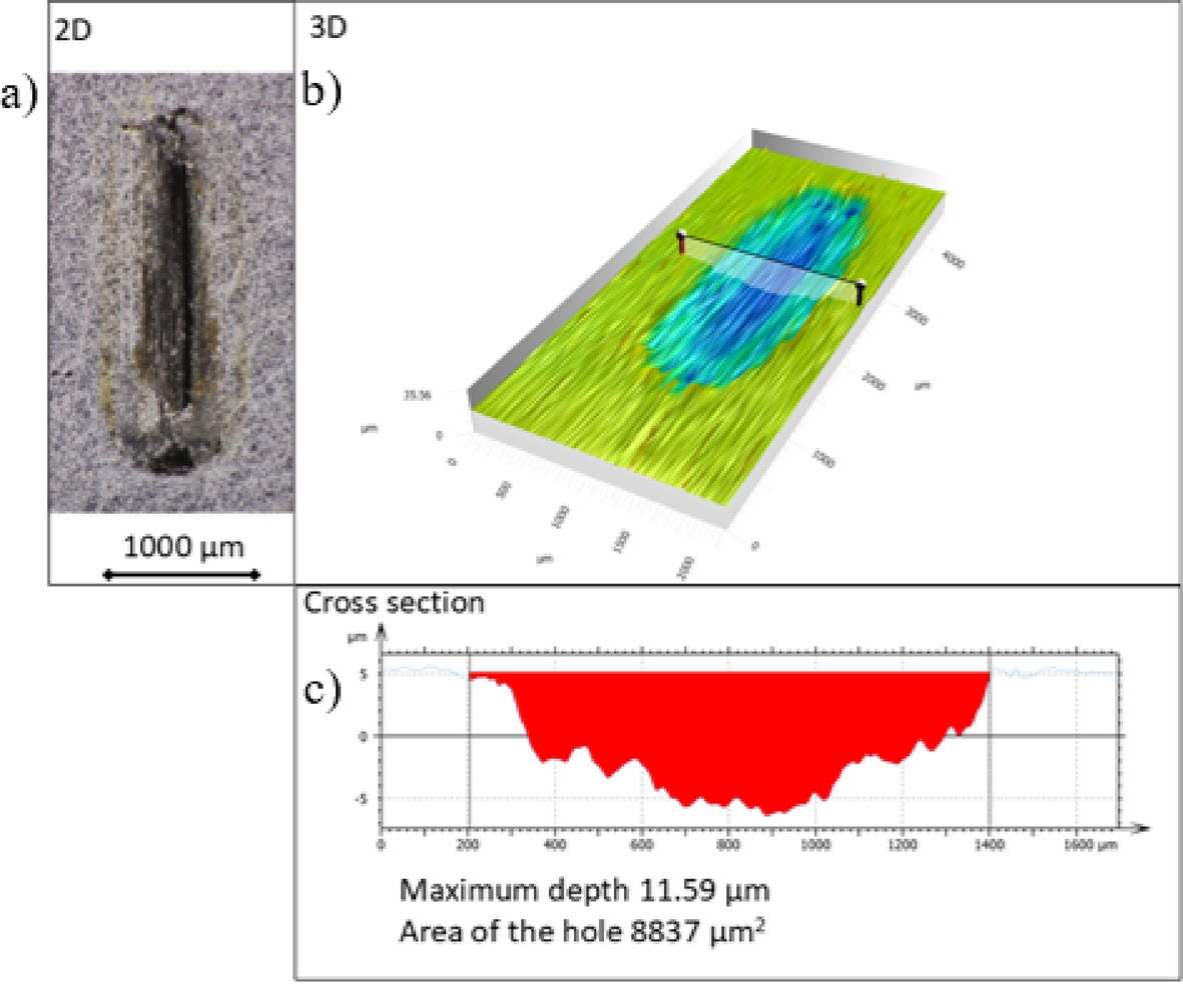
(**a**) 2D and (**b**) 3D profile of worn surface with (**c**) measured cross-sectional area at specific location.

After the reciprocal sliding tests, the dimensions of the wear tracks on individual discs samples were measured using a 3D digital microscope (Hirox HRX-01, HR-2500E, Magnification 0-2500 ×, resolution: 2.86 μm - horizontal, 50 nm- vertical), with nano-point scanner (NPS1, resolution: 1 μm-lateral, 20 nm-vertical; working Z range 150 μm). At first step the wear track at 50x magnification (resolution 2.86 μm) was positioned with the digital microscope (Fig. 1a). Than the nanopint scanner (sensitivity 150 nm, sensor control 200–1000 points /s) scans the worn volume with scanning speed 1200 μm /s  (Fig. 1b). The dimensions of the wear track and characteristic cross-sections were read on several characteristic locations along the wear track profile with Mountains^®^ 9 measurement software (Mountains Map)^44^, following the procedure established in earlier studies (Fig. 1c)^8^. The specific wear rate was calculated from the measured wear volume divided by the product of the normal load and the sliding distance.

After each test, the contact sliding surface on the steel ball was marked with an electric pen, to ensure that the wear track remains clearly defined and suitable for subsequent SEM analysis. With the digital microscope the shape and dimensions of the surface films on the steel ball were determined.

In addition, the topography of the worn discs and counter-ball surfaces was investigated using a scanning electron microscope (SEM; JEOL, JSM IT100, Tokyo, Japan) at an accelerating voltage of 15 to 20 kV. Also elemental analysis (energy dispersive spectroscopy - EDS) was performed on transfer films of counter-ball surfaces.

## Results and discussion

### Running in time

**Fig. 2:**
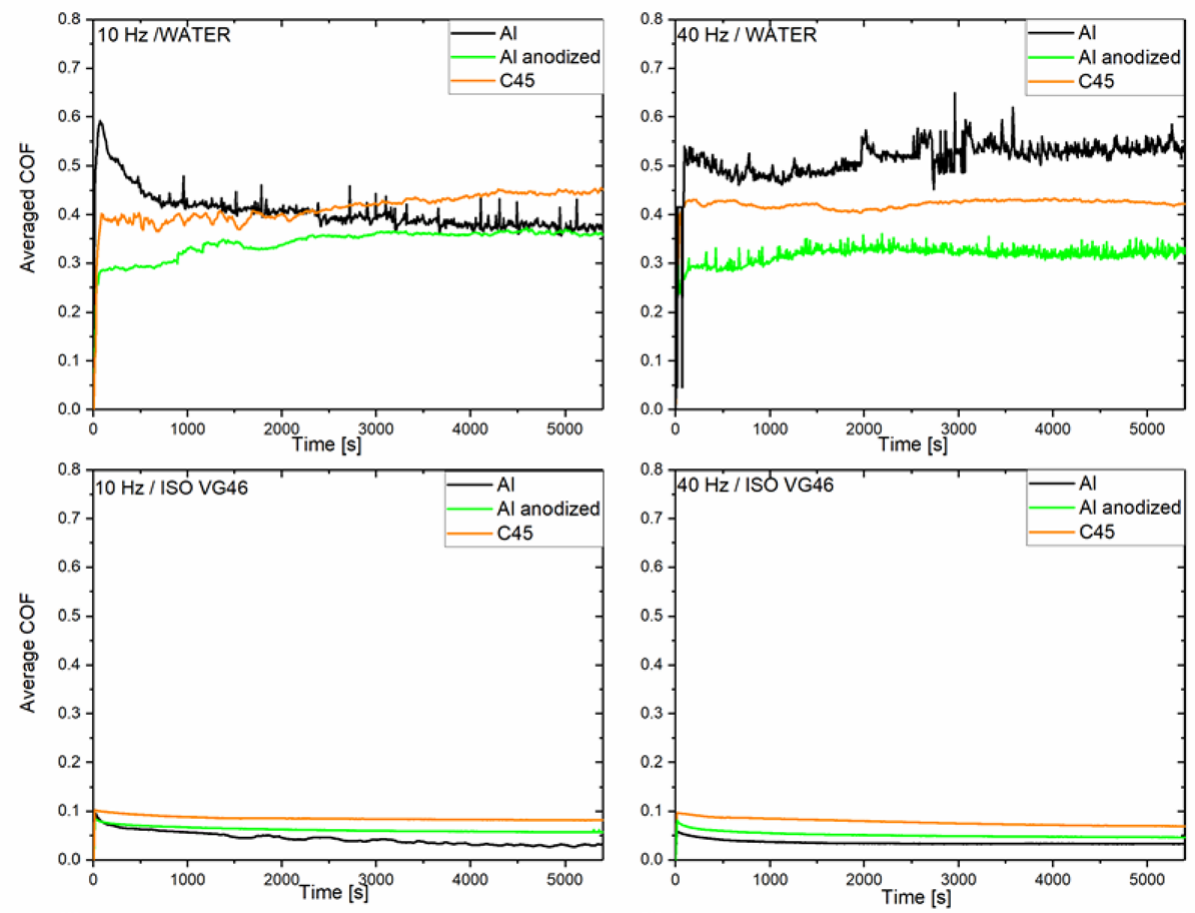
Evolution of coefficient of friction as a function of sliding time for individual tested material pairs in water and oil at both sliding speeds.

The course of the measured coefficient of friction for all tested materials at two different sliding velocities in both water and oil ISO VG46 are presented on Fig. 2.

In water for all tested materials, there was a short running in period, within the first 300 s, at both sliding speeds. The fluctuations in measured coefficient of friction were also evident. They are in the form of high-frequency noise, and are especially characteristic for natural aluminium alloy, even more predominant at higher sliding speed. Less intensive fluctuations are present even for nitrided C45 steel in water compared to oil lubricated conditions. They are probably the consequence of water not being able to provide good separating layer, causing the rough surface interactions and microstructural effects. The fluctuations indicate lack of stability despite lubrication^29^ .

At the beginning of the experiment there is slight increase in measured coefficient of friction, in case of natural aluminium in water, at lower sliding speed. Measured coefficient of fiction than decreases, around 40 % to reaching stable value. In case of anodized aluminium slight increase, around 20 % of coefficient of fiction was found in the running in period, before reaching steady value. Similar trend of coefficient of fiction change in natural aluminium alloy, compared to anodized aluminium alloy were found in earlier study under dry conditions^45^. Measured values of coefficient of friction and ratio natural/anodized are comparable to the earlier study of different pores size of anodized aluminium alloy^46^ (at sliding speed 0.011 m/s and 3.5–4.5 N normal load) in water. Slightly lower coefficient of friction measured after anodization is probably due to higher surface hardness of anodized alloy. The effect lower and more stable coefficient of friction after anodization is similar to earlier measurements of aluminium alloy 6061, due to increased surface hardness and formation of aluminium oxide layer^29^. In earlier study^46^, both samples (natural and anodized) approached comparable steady state coefficient of friction around 0.4. From the Fig. 2, it is evident that anodization smoothed the natural aluminium alloy response in water, especially at lower sliding speed.

In case of hydraulic oil, from the results, it is evident, that coefficient of friction reaches stable value relatively quickly after the begging of the test, within the first 25 s, regardless the sliding velocity or material. The only fluctuations are observed at lower speed for natural aluminium alloy, based on small periodic shape they indicate stick-slip behaviour due to adhesion and release cycles. The results obtained in oil are comparable to result of plasma electrolytic oxidation (PEO) and hard-anodized (HA) 6082 aluminium alloy at 0.1 m/s^47^, due to boundary lubricating film at the interface. In that study coefficient of friction reached stable but slightly higher value after HA (around 0.1) and after PEO (around 0.08), at higher normal loads (100 N) compared to our experiments with 50 N normal load. The results of this study are also comparable to coefficient of friction of PEO aluminium alloy Al6061 exposed to different environmentally acceptable lubricants ( COF ~ 0,1) with comparable viscosity as ISO VG 46 and comparable loading parameters (50 N, 50 Hz, at 80 °C, for 120 min).

### Coefficient of friction

**Fig. 3:**
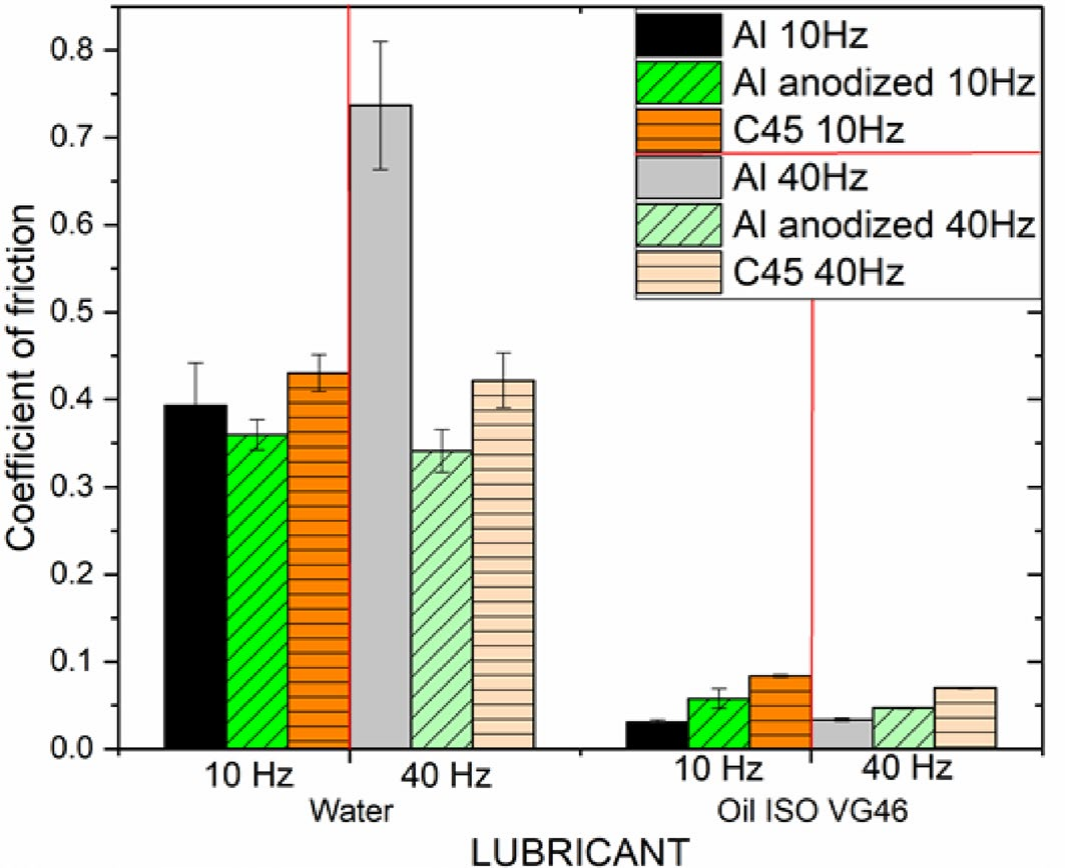
Average stable coefficient of friction in contact of individual tested material pairs in oil and water, at two different sliding speeds.

The average steady-state friction coefficients with standard deviations, for individual material pairs in water and oil ISO VG46, at both tested sliding speeds are shown in Fig. 3.

In water, the lowest steady-state coefficient of friction was measured between a steel bearing ball and anodized aluminium alloy EN AW-6082, and was not significantly affected by sliding speed (0.36 ± 0.02 at 10 Hz, and 0.34 ± 0.02 at 40 Hz respectively). The results showed that the coefficient of friction in the case of the natural aluminium alloy (0.39 ± 0.05 at 10 Hz, and 0.74 ± 0.07 at 40 Hz respectively) is higher than after standard anodization, at both sliding speeds. The difference is significant at higher sliding speed, since coefficient of friction of natural aluminium was 2.1. higher compared to anodized aluminium. In case of nitrided C45 steel, measured coefficient of friction was slightly higher than after standard anodization (0.43 ± 0.2 at 10 Hz, and 0.42 ± 0.03 at 40 Hz respectively), at both sliding speeds, and was not significantly affected by sliding speed.

In hydraulic oil, it is observed that the friction coefficient for all selected material pairs decreases significantly, compared to water lubricated conditions. In the case of the natural aluminium, the coefficient of friction in oil is 12.8 times lower (0.03 ± 0.03), in case of anodized aluminium alloy 6.2 times lower (0.06 ± 0.01) and in case of nitrided C45 steel about 5.1 times lower (0.08 ± 0.01) at lower sliding speed. At higher sliding speed measured coefficient of friction in oil slightly increased about 18 % for anodized aluminium EN AW-6082 and nitrided C45 steel, and was almost not affected by sliding speed for natural aluminium.

Based on the results of analyses of water and oil wettability of similar aluminium alloy (Al 6016)^48^, show that sandpaper-polished natural aluminium is slightly hydrophobic in water (contact angle ~ 91°) but strongly oleophilic (oil contact angle ~ 32°). Under comparable anodization conditions to our work (15 V, 18 °C), a one-step anodizing process creates a dimpled oxide surface that can act as a reservoir for lubricants. When these dimples are small, the anodized layer becomes both hydrophilic and oleophilic (water and oil contact angles < 90°). However, because the oxide film produced by standard anodizing is relatively thin, its ability to retain oil can be limited^48^. These characteristics help explain why natural and anodized aluminium behave differently in water and oil lubrication. In water, natural aluminium is poorly protected, and the unstable water film leads to high adhesion. In contrast, the hydrophilic anodic oxide supports a more stable water layer, reducing adhesion and resulting in a slightly lower friction coefficient. Under oil lubrication, the anodized surface—being rougher due to its porous oxide—creates more asperity contacts than the smoother ground natural aluminium surface, leading to a higher coefficient of friction.

From the results, we can conclude that the change in friction for both nitrided C45 steel and anodized aluminium alloy EN AW-6082 when hydraulic oil is switched with water is similar. Coefficient of friction decreases about 5–6 times, The decrease is not significantly affected by sliding speed. The most significant reduction in the measured coefficient of friction was observed for the natural aluminum alloy when comparing water-lubricated to oil-lubricated conditions. This indicates that both the lubricant type and the sliding speed have a pronounced influence on the frictional behavior of the untreated aluminum alloy.

### Specific wear

#### Wear track analyses

**Fig. 4:**
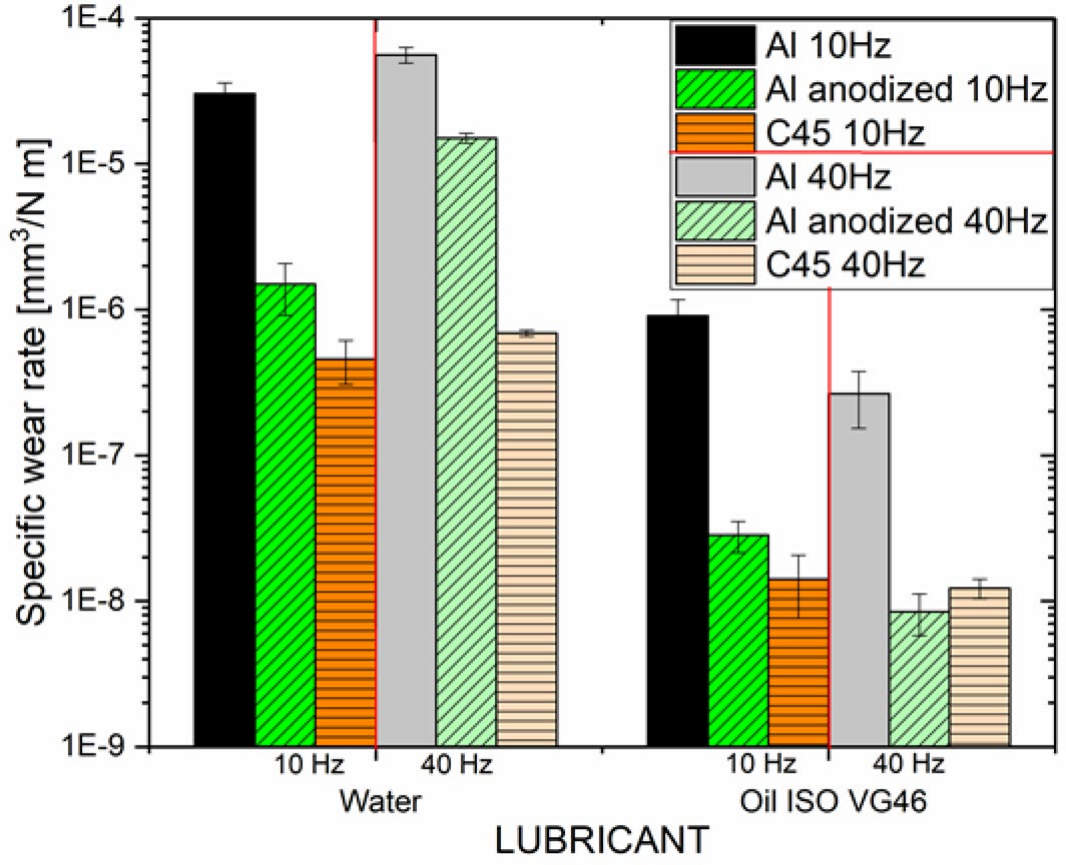
Average specific wear of individual materials in ISO VG46 oil and demineralized water, at two different sliding speeds.

The Hirox HRX-01 microscope with an additional nanodot confocal profilometer – NPS1 allowed us to capture the wear track and obtain its 3D profile. Based on the overall dimensions of the wear track and the cross-section measured along the 3D profile, the specific wear of discs made of individual materials in contact with a steel bearing ball was calculated, in the case of using water or oil ISO VG46 at both sliding speeds, which is shown on Fig. 4.

In water, the specific wear was the highest for natural aluminium alloy EN AW-6082 at both sliding speeds (3.04 × 10^− 5^ mm^3^/Nm up to 6 × 10^− 5^ mm^3^/Nm). After standard anodization, specific wear was lower, under all tested conditions, compared to natural aluminium alloy. Measured specific wear for anodized aluminium alloy EN AW-6082 in water, was almost an order of magnitude lower (1.49 × 10^− 6^ mm^3^/Nm) at lower sliding speed. However, measured specific wear was only slightly lower at higher loading speed (1.49 × 10^− 5^ mm^3^/Nm) compared to natural aluminium alloy. Measured values od anodized alloy specific wear were slightly higher compared to the specific wear measured for nitrided C45 steel (6.34 × 10^− 7^ mm^3^/Nm), at lower sliding speed. However, both aluminium samples had two orders of magnitude higher specific wear compared to the specific wear measured for nitrided C45 steel (6.9 × 10^− 7^ mm^3^/Nm).

When hydraulic oil ISO VG46 was used instead of water, the specific wear, similarly as the measured coefficient of friction, decreases in all cases. At both sliding speeds, the specific wear was highest for the natural aluminium alloy EN AW-6082 (9.07 × 10^− 7^ mm^3^/Nm and 2.65 × 10^− 7^ mm^3^/Nm respectively), which is almost 33 times lower compared to water lubricated conditions. The highest decrease in specific wear occurs for the anodized aluminium alloy EN AW-6082, almost 52-fold decrease at lower speed (2.83 × 10^− 8^ mm^3^/Nm) and 1771-fold decrease (8.5 × 10^− 9^ mm^3^/Nm) at higher sliding speed, compared to water lubricated conditions. In oil anodized aluminium showed closer vales of specific wear to the specific wear measured for nitrided C45 steel (around 1.3 × 10^− 8^ mm^3^/Nm at both siding speeds). In the oil, higher sliding speed lowered measured specific wear for all tested materials, but the difference was not significant. Measured values of specific wear are comparable to measured specific wear of anodized and PEO aluminium alloy tested under different environmentally acceptable lubricants (with viscosity comparable to hydraulic oil ISO VG46), around 6.5 × 10^− 8^ mm^3^/Nm, at 50 N normal load and 50 Hz frequency^28^. Measured values od specific wear are considerably lower compared to the measurements of HA and PEO treated aluminium in oil at 50 N, and 0.1 m/s, around x 10^− 6^ mm^3^/Nm ^47^. However, in these experiments two times smaller sliding ball was used, thus higher disc wear could be expecting, compared to our experiments.

#### Wear surface analyses

**Fig. 5:**
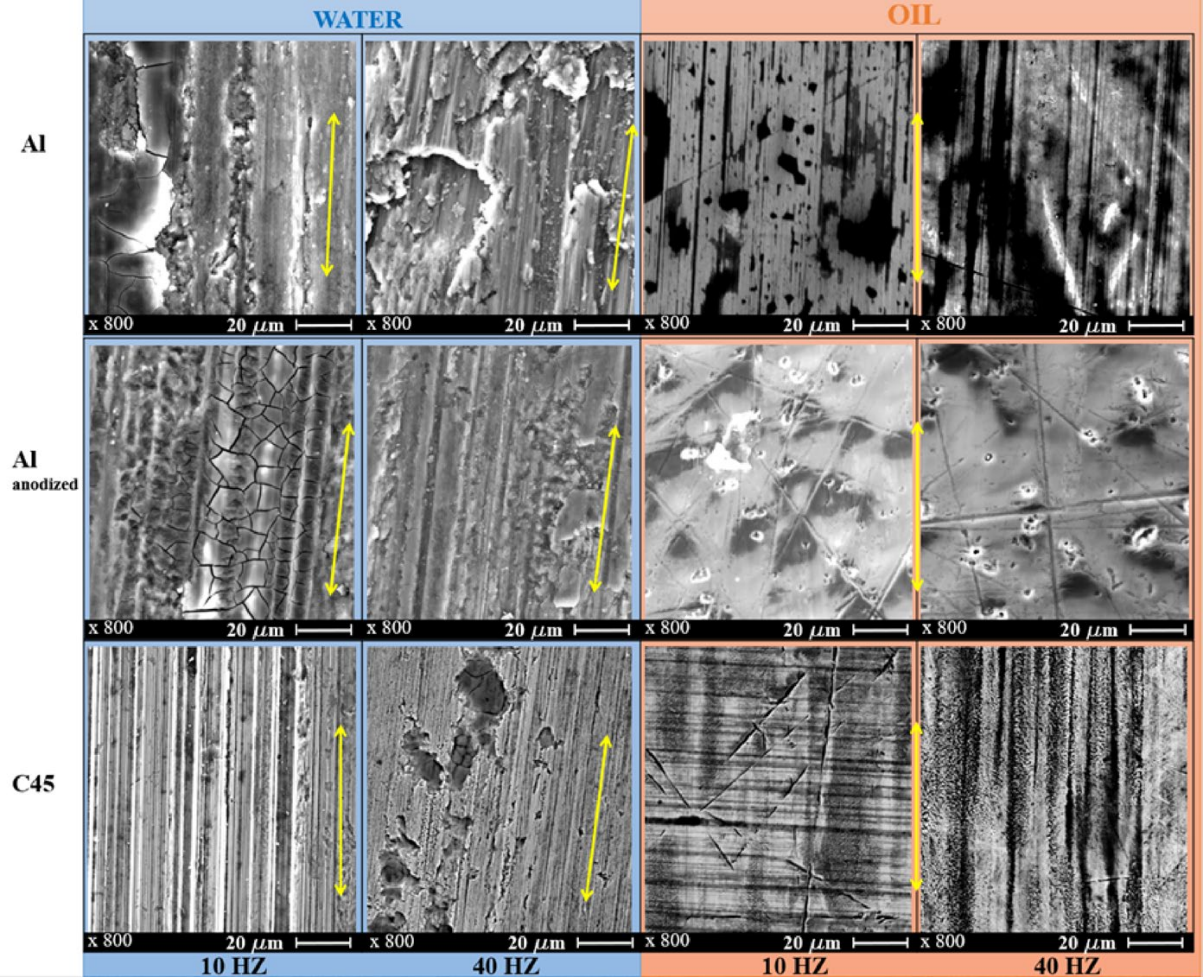
SEM micrographs of worn surfaces in water and oil at both sliding speeds. *yellow arrow indicates the sliding direction.

SEM images of worn surfaces (Fig. 5) help to indicate similarities and differences in wear mechanisms of natural aluminium and anodized aluminium compared to C45 when lubricated with water or common hydraulic oil ISO VG46, at two different sliding speeds.

In water intensive long abrasive grooves parallel to the sliding direction are evident for all materials, even at lower sliding speed.

In case of natural aluminium alloy beside present cracks, wide, deep and irregular grooves are predominant showing poor water lubrication and high abrasion. At higher sliding speed the wear mechanism changes. Severe ploughing and material smearing with wear debris are noticeable. There is superimposed adhesive wear on abrasion. Worn fragments adhere along entire wear track, similarly as in earlier study of similar aluminium alloy Al 6061^29^.

In case of anodized aluminium alloy there are much finer grooves compared to natural aluminium alloy. Cracked oxide pattern is typical for anodized layers but does not indicate failure, at lower sliding speed. The porous anodic layer appears cracked but reduces large-scale ploughing. Scratches are shallower and narrower, indicating lower adhesion and more stable water film compared to natural aluminium. These adhesive smearing fragments are smaller and less prominent in anodized aluminium compared to natural aluminium, indicating that the protective anodized layer is steel present, even at higher sliding speed, similarly as for aluminium alloy 6061 under lower loads^29^. The cracks of oxide layer are similar to cracks observed for same aluminium alloy 6082^20^ after hard anodization, and 6060^18^ after innovative hard anodization but under dry conditions. The cracks of anodized aluminium are also in consistence with porous aluminium alloy in water, at significantly lower loads^46^.

In case of nitrided C45 steel, uniform abrasive grooves show stable sliding track with minimal debris, at slower sliding speed. At higher sliding speed, deeper but still more uniform and stable abrasion, with some micro-ploughing is observed.

For comparison reason, if we look at the worn micrographs in hydraulic oil (Fig. 5), only small stretches in sliding direction are present in natural aluminium alloy and nitrided C45 steel at higher sliding speed. In natural aluminium grooves remain visible but appear narrower and darker, suggesting partial hydrodynamic film formation. Some parallel tracks appear smoother compared to water, indicating reduced direct contact. Oil provides some lubrication, preventing large debris formation.

However, anodized layer is good in protecting the material, at both sliding speeds since only small bursts are present on oxide layer. The anodized aluminium alloy in oil exhibit numerous scattered pores and crater-like features, which likely come from local micro-fracture or separation of the porous anodic oxide. Although the wear grooves are less pronounced than under water lubrication, the surface displays a rough and irregular micro-topography associated with pore collapse and localized brittle damage. The observed scratches indicate predominantly abrasive wear, accompanied by fragmentation of the non-uniform oxide layer. Compared to the water-lubricated condition, the oil-lubricated tracks show increased pitting and surface irregularity. This behaviour is consistent with the poor wettability of oil on anodized oxide, which results in weaker lubrication, higher friction (as shown in Figs. 2 and 3), and a greater tendency for oxide fracture. The particle-like features on the anodized aluminium wear track correspond to debris originating from the anodic oxide layer itself. These deposits most likely represent detached fragments of the brittle, porous oxide. During sliding, shear stresses promote flaking and spallation of the oxide, and due to the limited dispersion of debris in oil, these fragments accumulate on the wear track as irregularly shaped incrustations.

#### Transfer on sliding counterpart in water

**Fig. 6:**
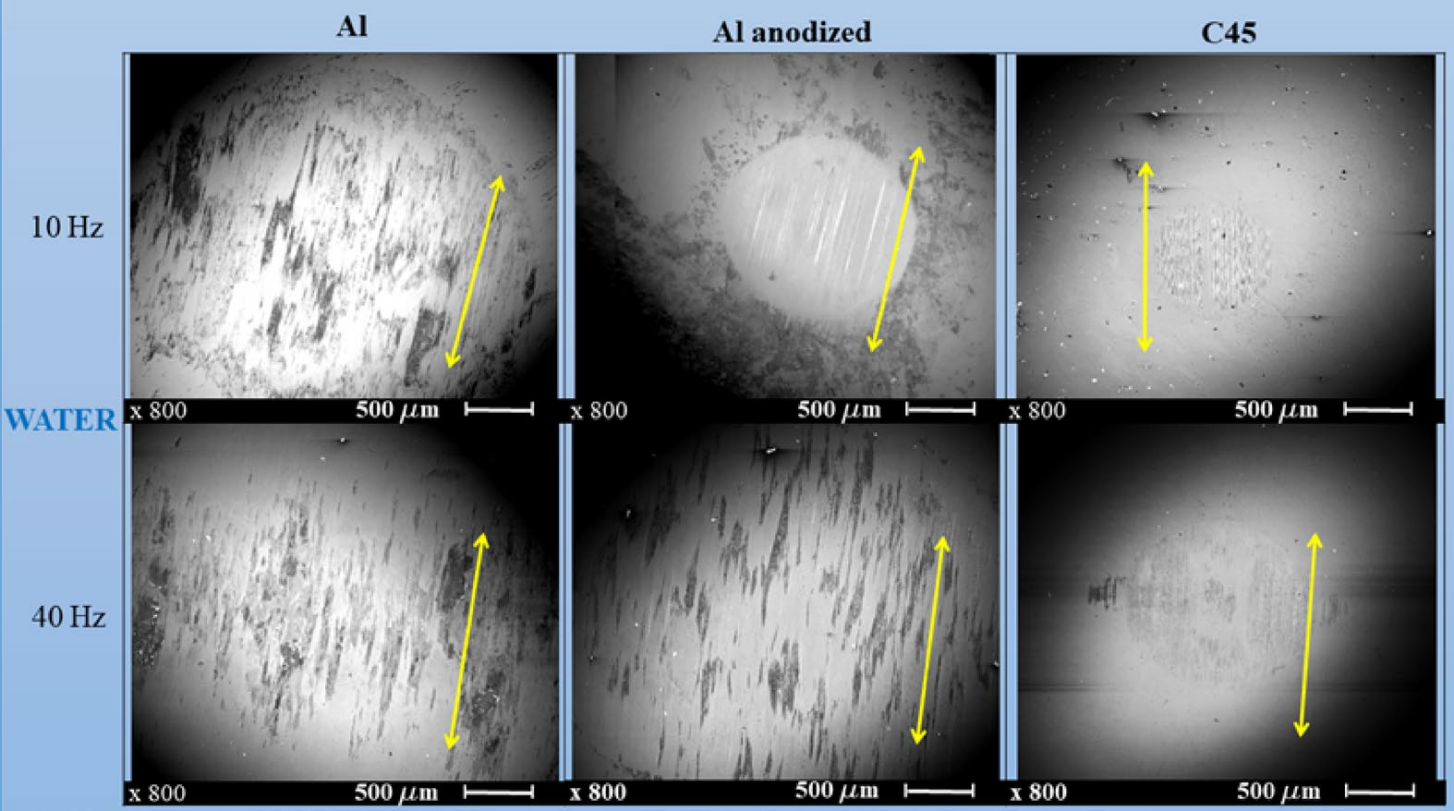
SEM micrographs of the transfer on counter stainless-steel balls in water *yellow arrow indicates the sliding direction.

Transfer film was only observed in water. On the Fig. 6 the observed transfer on counter stainless-steel balls are presented. At lower sliding speed, the smallest coverage area was measured for C45 (0.67 mm^2^). The measured coverage area was 2.2 times bigger after sliding against anodized aluminium alloy (1.48 mm^2^). The measured coverage area was 10 times bigger for natural aluminium alloy (6.88 mm^2^).

At higher sliding speed, transfer film become more intense and coverage area increased for all tested materials, with ~ 2 times increase for both nitrided C45 steel and natural aluminium alloy, and ~ 6 times increase for anodized aluminium alloy. The highest coverage area was again observed for natural aluminium alloy (14.5 mm^2^). The anodization process slowed down the transfer process as compared to natural aluminium alloy, and in this case, measured coverage area was 9.1 mm^2^.

The transfer observed after sliding against nitrided C45 steel at both sliding speeds was similar with the shape and coverage area to the transfer film observed after sliding against anodized aluminium alloy at lower sliding speed. There were characteristic thin scratches in the sliding direction.

In case of natural aluminium alloy, beside bigger coverage area and transfer along the sliding direction also some thick zones of transferred and pasted material were present. These dark regions were also present in case of anodized aluminium at higher sliding speed. In case of anodized aluminium alloy these regions of intense transfer were equally dispersed over the coverage area. In case of natural aluminium alloy, some thicker and bigger transfer film areas are in the central zone.

**Fig. 7:**
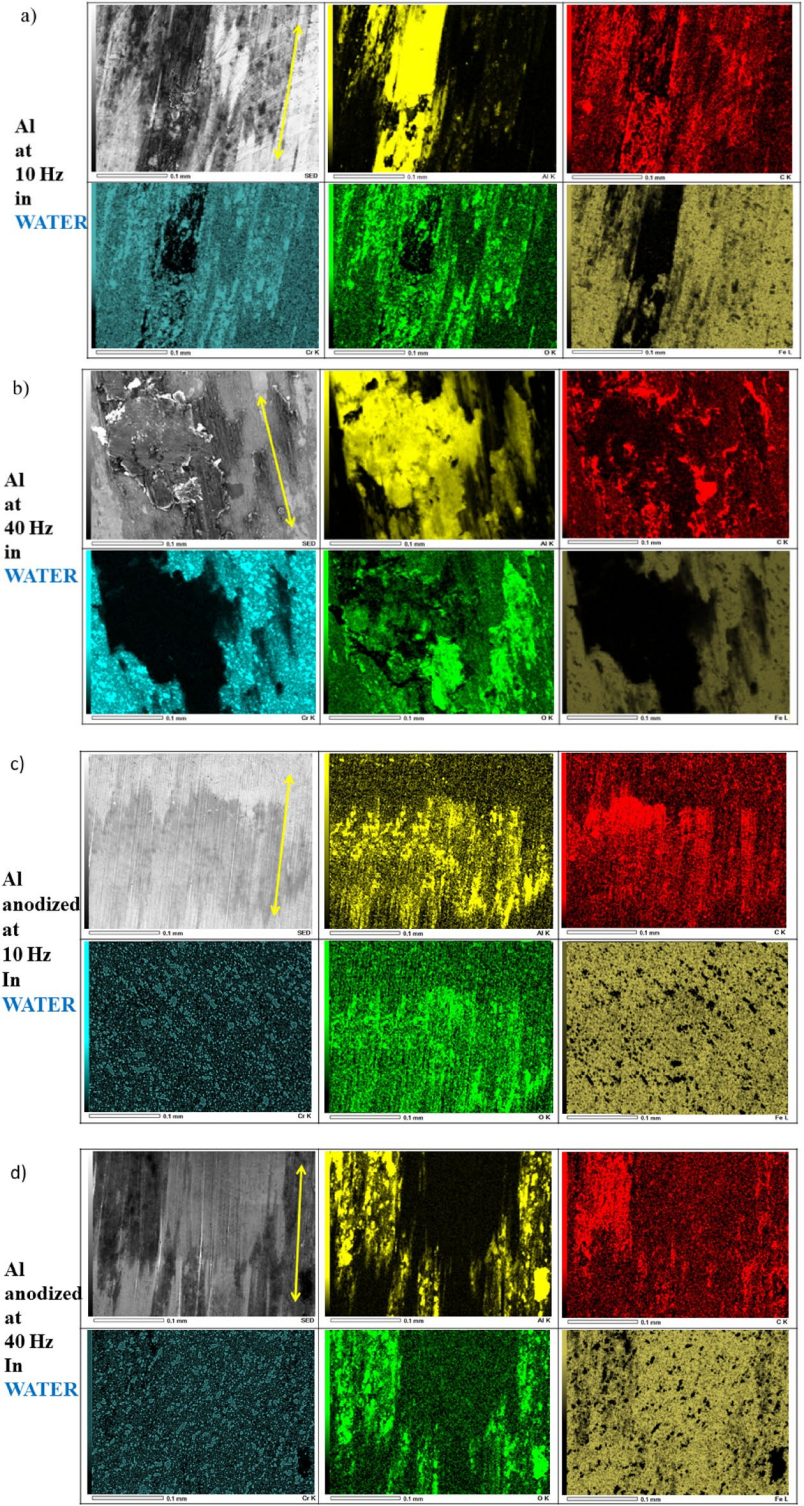
EDS micrographs of transfer on counter stainless-steel balls in water after sliding against aluminium and anodized aluminium at both sliding speeds *yellow arrow indicates the sliding direction.

With the EDS analyses (Fig. 7), we have mapped the elements present in the transfer film, and differences in spatial distribution of elements across counter steel ball after sliding against anodized and natural aluminium alloy at both sliding speeds. In general, less countersurface transfer material was observed, compared to dry sliding experiments of hard anodized aluminium alloy^18,20^.

In case of natural aluminium alloy, there was clear evidence of strong continuous aluminium and thin, discontinuous oxide film on lower sliding speed (Fig. 7a). Transfer particles are large and irregular, since lower sliding speed promotes adhesive lump formation. Oxygen distribution is patchy, indicating localized oxidation. Wider and thicker debris regions of aluminium were present at higher sliding speed, indicating severe adhesive wear (Fig. 7b). Debris is coarser and more compacted due to dynamic rolling under high-frequency contact. Also, thicker, compacted aluminium oxide debris are present probably due to higher local temperature in water and faster oxidation.

In case of anodized aluminium, at lower speed some small zones of scratch like patterns in the sliding direction of aluminum and oxides were present (Fig. 7c). However, much more homogeneous and weaker compared to natural aluminium alloy, suggesting that anodic layer shields the undelaying aluminium alloy. Anodic oxide reduces adhesion by acting as a hard, chemically inert barrier. Transfer material is minimal, dominated by very small oxide fragments rather than large adhesive lumps. Scratches are shallow, narrow, because anisotropic ploughing is suppressed by the hard oxide. At lower sliding speed, mild abrasion is present. At higher sliding speed, there are still some debris zones of aluminium and oxides fine grooves, slightly more oxide cracking are present and some smaller particles compared to natural aluminium alloy (Fig. 7d). This indicates, that in case of anodized aluminium alloy, protective oxide layer, oxide layer is effective. Anodizing suppresses adhesive wear, reduces transfer, stabilizes oxide chemistry, and controls debris size. At higher speed, some particles and debris are present, with intensity and coverage smaller compered to natural aluminium. This is indicating that protective layer is being slowly degraded. While anodizing improved performance of natural aluminium significantly, micro-cracking’s and oxide spalling at higher sliding speed indicate limitations of standard anodizing compared to micro-arc oxidation coating on aluminium alloy^17^ *.*

## Conclusions

This study investigated the tribological behaviour of natural and anodized EN AW-6082 aluminium alloys in water, sliding against stainless steel at two different speeds. The experimental results were compared to the results with standard hydraulic-valve material - lubricant combination: nitrided C45 steel and hydraulic oil ISO VG46. The findings demonstrate that anodizing significantly improves the tribological performance of aluminium alloy and enables its potential use as an alternative material for hydraulic valve components even in harsh water lubricated conditions.

- When lubricated with hydraulic oil, the anodized aluminium alloy exhibited excellent tribological performance, with a very low coefficient of friction (0.06) and low specific wear (2.83 × 10⁻⁸ mm³/Nm), comparable to nitrided C45 steel. The anodic oxide layer effectively protected the base material at both sliding speeds, confirming its suitability for oil-lubricated hydraulic applications.
- Across all materials, the transition to water lubrication resulted in a substantial increase in friction and wear, accompanied by the formation of transfer films on the stainless-steel counter surface. Sliding speed emerged as a critical factor governing wear mechanisms and transfer behaviour.
- At lower speed, anodizing significantly improved the sliding performance of aluminium alloy in water. The anodized alloy showed smoother friction behaviour with reduced fluctuations, lower wear than the natural alloy, and specific wear values comparable to nitrided C45 steel. SEM observations revealed only minor cracking and shallow scratches of the oxide layer, while EDS confirmed limited, scratch-like transfer of aluminium and oxides. In contrast, the natural alloy showed severe grooving and intense material transfer.
- At higher speed, the protective performance of the anodized oxide layer diminished. Although the anodized alloy still produced the lowest friction coefficient among all tested materials (0.34) and lower transfer than the natural aluminium alloy, its wear significantly increased and exceeded that of nitrided C45 steel. SEM and EDS analyses indicated pronounced oxide-layer damage, wear debris, and substantial material transfer to the counter surface, indicating partial breakdown of the anodic coating.
- Overall, the results show that anodized EN AW-6082 aluminium alloy is a viable material for sliding contacts in hydraulic valves, particularly under oil lubrication and under moderate water-lubricated conditions where sliding speeds are low. However, at higher sliding speeds in water, the anodic layer may fail, increasing the risk of wear and material transfer. To ensure suitability under more demanding water-lubricated conditions, surface-enhancement strategies—such as hard anodizing, thicker oxide layers, or extended oxidation treatments—should be explored to improve durability and corrosion resistance. Future work should also examine the influence of normal load, temperature, and transfer-film composition to better understand wear mechanisms and optimize coating performance.

## Data Availability

The datasets used and analysed during the current study are available from the corresponding author upon reasonable request.
